# Contextual diversity facilitates learning new words in the classroom

**DOI:** 10.1371/journal.pone.0179004

**Published:** 2017-06-06

**Authors:** Eva Rosa, José Luis Tapia, Manuel Perea

**Affiliations:** 1 Universidad Católica de Valencia, Valencia, Spain; 2 Universitat de València, Valencia, Spain; 3 BCBL, Basque Center on Cognition, Brain, and Language, Donostia, Spain; Kyoto University, JAPAN

## Abstract

In the field of word recognition and reading, it is commonly assumed that frequently repeated words create more accessible memory traces than infrequently repeated words, thus capturing the word-frequency effect. Nevertheless, recent research has shown that a seemingly related factor, contextual diversity (defined as the number of different contexts [e.g., films] in which a word appears), is a better predictor than word-frequency in word recognition and sentence reading experiments. Recent research has shown that contextual diversity plays an important role when learning new words in a laboratory setting with adult readers. In the current experiment, we directly manipulated contextual diversity in a very ecological scenario: at school, when Grade 3 children were learning words in the classroom. The new words appeared in different contexts/topics (high-contextual diversity) or only in one of them (low-contextual diversity). Results showed that words encountered in different contexts were learned and remembered more effectively than those presented in redundant contexts. We discuss the practical (educational [e.g., curriculum design]) and theoretical (models of word recognition) implications of these findings.

## Introduction

One of the most studied phenomena in the field of visual word recognition and reading is the word-frequency effect: response times in word recognition experiments (e.g., lexical decision, naming, semantic categorization) and eye fixation durations in sentence reading experiments are shorter for words that are frequently encountered than for words that are infrequently encountered (see [1] for review; see also [2], for the first demonstration of the effect). Indeed, word-frequency plays a critical role in models of visual word recognition [3–5] and in models of eye movement control in reading [6–7].

Although none of the above-cited models has a mechanism to create new lexical representations, the implicit assumption is that the source of the word-frequency effect rests on the principle of mere repetition: Items that are encountered more frequently create more accessible memory traces than those items that are rarely encountered. However, recent findings have questioned the role of mere repetition in the accessibility of words. Adelman, Brown, and Quesada [8] found that, in skilled adult readers, contextual diversity (defined as the number of different contexts [e.g., films, books] in which a word appears) was a better predictor than word-frequency in various word recognition tasks (see also [9–14]). Likewise, Plummer, Perea, and Rayner [15] extended the contextual diversity effect to eye fixation times during sentence reading: fixation durations were shorter for words with high-contextual diversity than for words with low-contextual diversity.

Although contextual diversity and word-frequency seem to be highly associated (i.e., words that appear in many contexts also tend to be very frequent words), the two effects may be different in origin. Specifically, Vergara-Martínez, Comesaña, and Perea [16] reported that while both contextual diversity and word-frequency have a facilitative role when measuring lexical decision times, the mechanism underlying this behavioral facilitation seems to differ. The electrophysiological counterpart of word-frequency consisted of a smaller N400 amplitude for high than for low-frequency words—this can be interpreted in terms of facilitative lexical retrieval. In contrast, the electrophysiological counterparts of contextual diversity consisted of larger N400 amplitudes for high- than for low-contextual diversity words—this can be interpreted in terms of greater semantic richness. Therefore, encountering words in different contexts may lead to semantically richer representations.

Notably, there is empirical evidence that shows the importance of contextual diversity when learning new words. Kachergis, Yu, and Shiffrin [17] carried out three experiments using the cross-situational word-learning paradigm introduced by Yu and Smith [18]. In each trial of the training phase, participants were presented with four novel objects and their corresponding spoken nouns (pseudowords). Kachergis et al. [17] manipulated frequency of occurrence (number of times that a pair was repeated within the same set of trials) and contextual diversity (number of other stimuli a given pair coincides with). After the training phase, participants had to choose the appropriate object for each noun out of eighteen potential objects. They found better performance (i.e., a learning advantage) for the pairs presented with high contextual diversity (i.e., those that co-occur with many different pairs during training) relative to those with low contextual diversity. The importance of contextual diversity when learning words has also been observed using a correlational methodology. Hills, Maouene, Riordan, and Smith [19] found that a word’s contextual diversity—defined as the number of unique word types a word co-occurs with in caregiver speech in the CHILDES database [20]—predicted the order of word learning at an early age: the earliest learned words were also the most contextually diverse in the learning environment.

In a recent word-learning study, Jones, Johns, and Recchia [21] argued that the semantic distinctiveness across different contexts—rather than mere number of contexts—could determine successful word learning. To measure semantic distinctiveness, they quantified the similarity of a pair of documents as the proportion of semantic information shared by both contexts (i.e., redundant information). They then defined the semantic distinctiveness of a word as the mean dissimilarity (i.e., 1- similarity) over all the documents in which the word occurs. Johns et al. ([21] Study 1) found that holding constant the number of different documents in which a word appears in a corpus, lexical decision times were faster for those words that occurred in more semantically distinct contexts than for those that occurred in redundant contexts. To provide evidence for a causal role of contextual diversity in word learning, Jones et al. ([21] Study 2) conducted an artificial language learning experiment. This allowed them to independently manipulate document count [word frequency] and semantic distinctiveness. During the training phase, subjects were presented a set of 450 slides, each of which contained an image along with a 3-word sentence meant to describe the object. They manipulated the number of contexts in which a target stimulus appeared (i.e., document count) and the semantic distinctiveness of these contexts (the target was inserted in the same sentence and with the same reference image vs. the target was inserted in different sentences with different reference images). For targets appearing in a large number of contexts, Jones et al. ([21] Study 2) found shorter response latencies in a pseudolexical decision task (“Was this a word in the language you just learned?”) when these contexts were high in semantic distinctiveness than when they were low. However, targets appearing in a large number of redundant contexts produced equivalent response latencies as the words that appeared in a lower number of redundant contexts. Jones et al. [21] concluded that contexts that provide redundant information with past experience are not encoded with the same intensity as those that provide unique information, and thus, they do not facilitate learning as much. To explain these results, Jones et al. [21] proposed the Semantic Distinctiveness Model. This model introduces a mechanism that gauges the information redundancy between the current context and the word’s current memory representation. The memory strength of a word increases when there is a detectable modification in context (i.e., more diverse contexts would produce a more accessible representation).

More recently, Johns, Dye, and Jones [22] extended this line of research to a more ecological scenario. In the training phase, university students read small fragments extracted from articles, books, and newspapers in which low-frequency words had been replaced by pronounceable nonwords (i.e., target stimuli). The participants’ task was to read the texts and evaluate on a 7-point scale to what extent they understood each passage. Target stimuli were presented five times in two experimental conditions: high contextual diversity (five fragments from highly distinctive contexts) and low contextual diversity (five redundant inserted fragments). After reading the texts, participants performed a pseudolexical decision task—this involved recognizing the targets presented during the training phase—and a semantic similarity judgment task in which each of the studied items was paired with four close associates and subjects were asked to rate how similar each pair was in meaning on a scale from 1 to 7. Johns et al. [22] found that participants recognized faster and more accurately the newly acquired stimuli when they appeared in highly distinctive contexts than when they were presented in redundant contexts. However, in the semantic judgment task, subjects rated items trained in the low diversity condition as significantly more similar to their four closest associates, as those trained in the high diversity condition. Johns et al. [22] concluded that redundant contexts resulted in more stable semantic representations. While the findings from Johns et al. [22] are undoubtedly important, high and low contextual diversity words in their experiment differed not only in the number and semantic distinctiveness of the different contexts in which the word target appeared, but also in the meaning of the words. For example, when the word *constellation* was included in the high contextual diversity condition, it could refer to stars, symptoms of a disease, or freckles. Therefore, the Johns et al. [22] study cannot be used to disentangle the effects of contextual diversity from the effects of semantic richness of the learned semantic representations.

The main aim of the current experiment was to examine the role of contextual diversity in the acquisition of new words in a naturalistic environment, namely, while primary school students read texts during their regular classes. To this purpose, we directly manipulated the contextual diversity of a set of newly learned words. As in the Johns et al. [22] experiment, contextual diversity was operationalized as the number of highly distinctive (non-redundant) contexts in which a new word appears. Specifically, participants were asked to read texts about several topics, related to the content of three classes: Spanish language, Natural Sciences, and Mathematics. The new words could appear in different contexts/topics (high-contextual diversity) or only in one of them (low-contextual diversity). In all cases, each new word was presented three times (i.e., the frequency of the newly learned words was held constant in the high- and low-contextual diversity conditions). It is important to stress here that the meaning of the new words was kept constant in all the contexts. Therefore, the obtained effects would be attributed to contextual diversity and not to the words’ intrinsic semantic properties.

A key novel element of the present research is external validity. Unlike previous experiments that tested the effect of contextual diversity when learning new words in a lab environment with university students (e.g., [17, 21, 22]), the current experiment was conducted at the school, while children were learning new words in the classroom (i.e., a very ecological scenario). The effect of contextual diversity was measured in two memory tasks (free recall and recognition), a task that required matching words with pictograms (see [17] for a similar procedure with pictograms), and a multiple-choice test that required the completion of sentences by selecting the target words out of three lexical distractors (orthographically or phonologically similar)—note that this measures not only the acquisition of the meaning but also the correct orthography of target words. Finally, unlike Johns et al. [22], we did not employ a pseudolexical decision task because the number of target words per condition was too low to produce stable mean response times in a children population.

## Materials and methods

### Participants

The participants were 43 third-grade children from a middle-class public school in Spain. This study was approved by the Experimental Research Ethics Committee of the University of Valencia (Spain). We obtained written informed consent from their parents before participating in the experiment. The average age was 9 years (range: 8–9 years). Ten of the initial 43 participants were excluded from the final sample due to different reasons: two of them showed previous learning difficulties (attention deficit disorder and visual field defects), one of them did not have the parent's consent, and the remaining seven missed some of the experimental sessions. Of the remaining 33 participants, 20 were boys.

### Materials

We selected a set of 12 target words in Spanish (average length: 7.5 letters, range: 6–11), of which 11 were nouns and one was an adjective. These words did not occur in the LEXIN primary school lexical database in Spanish [23] and had a very low frequency of use (mean = 0.15, range 0–0.9) in the Spanish subtitle database [12]. To verify that these words were (normally) unknown by Grade 3 children, we recruited a different representative sample of Grade 3 children (N = 20) and asked them about the meaning of these words. None of them knew those words. The rationale for testing the target words on a different representative sample was that we did not want the experimental subjects to have any experience with the words they had to learn prior to the experiment. The list of target words in Spanish and English is presented in Appendix A in S1 File.

We created two counterbalanced sets of materials so that each word could appear in a high or a low diversity context. Each set consisted of 9 short texts (18 texts in total), equal in length (155 words) and difficulty, and appropriate for the reading level of the participants: 6 texts were short stories or fables, 6 were expository texts with natural or social science contents, and 6 were composed of simple math exercises. In Set 1, 6 of the target words appeared in a high diversity context (they appeared inserted in the three types of texts), and the remaining 6 words appeared in a low diversity context (they appeared only in one of the three types of text), whereas the opposite procedure was employed in Set 2. Four different target words were inserted in each text, making sure that each target word appeared just once in every text (always with the same gender and number, and with a stable meaning). In addition, in order not to reduce diversity, the same target words never coincided in different texts in the high diversity condition. All the target stimuli were concrete words with unambiguous meanings for adults. In addition, the three types of texts were constructed in such a way as to create a contextual constraint that would allow participants to derive the meaning of the new word, without the need of an explicit description. For example, for the word *batrachians*, the texts in both high and low diversity conditions contained clues about some characteristics of these animals: they move jumping, they inhabit ponds, they croak, or they eat insects (i.e., the inferred semantic representations were, on average, equally similar between the two conditions). Here is an example of how the word *batrachians* was inserted in three sentences from the three types of text in the high diversity condition. Fable: “Un pequeño grupo de *batracio*s bajaba saltando por un promontorio, de camino a una charca…” (“A small group of *batrachians* were jumping down a promontory, on their way to a pond…”) Science text: “…También hay animales, como los *batracios*, que se alimentan de otros animales de su ecosistema, como los insectos…” (“…There are also animals, such as the *batrachians*, which feed on other animals in their ecosystem, such as insects…”) Math problems: “Croando junto a una charca había 14 *batracios*…” (“Croaking next to a pond there were 14 *batrachians*…”) Appendix 1 shows another example of the three complete texts in which the word *forage* was inserted. Tables 1 and 2 can be a clarifying outline of how the target words were inserted into the different texts of sets A and B, respectively. At the end of each of the texts, we added two reading comprehension questions with three possible response options, of which only one was correct—the purpose was to ensure that the students were reading the texts comprehensively.

**Table 1:** Distribution of target words in the texts of the experimental set A.

**Fable 1**	**Fable 2**	**Fable 3**
Bermejo	Dehesa	Caninos
Batracios	Venado	Raigón
Promontorio	Promontorio	Promontorio
Guijarros	Guijarros	Guijarros
**Science text 1**	**Science text 2**	**Science text 3**
Caninos	Bermejo	Dehesa
Venado	Raigón	Batracios
Forraje	Forraje	Forraje
Simientes	Simientes	Simientes
**Math problems 1**	**Math problems 2**	**Math problems 3**
Dehesa	Caninos	Bermejo
Raigón	Batracios	Venado
Valvas	Valvas	Valvas
Vulpeja	Vulpeja	Vulpeja

Note: Target words belonging to the high contextual diversity condition: Bermejo, Caninos, Dehesa, Raigón, Venado y Batracios. Target words belonging to the low contextual diversity condition: Promontorio, Forraje, Valvas, Guijarros, Simientes y Vulpeja.

**Table 2:** Distribution of target words in the texts of the experimental set B.

**Fable 1**	**Fable 2**	**Fable 3**
Promontorio	Valvas	Forraje
Vulpeja	Simientes	Guijarros
Bermejo	Bermejo	Bermejo
Raigón	Raigón	Raigón
**Science text 1**	**Science text 2**	**Science text 3**
Forraje	Promontorio	Valvas
Simientes	Guijarros	Vulpeja
Caninos	Caninos	Caninos
Venado	Venado	Venado
**Math problems 1**	**Math problems 2**	**Math problems 3**
Valvas	Forraje	Promontorio
Guijarros	Vulpeja	Simientes
Dehesa	Dehesa	Dehesa
Batracios	Batracios	Batracios

Note: Target words belonging to the high contextual diversity condition: Promontorio, Forraje, Valvas, Guijarros, Simientes y Vulpeja. Target words belonging to the low contextual diversity condition: Bermejo, Caninos, Dehesa, Raigón, Venado y Batracios.

### Evaluation instruments

To assess the acquisition of the newly learned words, we employed four tasks: 1) a free recall task; 2) a recognition task; 3) a multiple-choice test with lexical distractors (orthographically or phonologically similar to the target word); and 4) a task that required matching words with pictograms. There was no time deadline for any of the tasks. The free recall task required participants to write down all the new words they had learned when reading the texts during the three training sessions. For the recognition test, we used a total of 54 synonyms of words extracted from the texts that constituted the experimental material—none of these words appeared in the LEXIN primary school lexical database in Spanish [23]. These 54 words were randomly presented to the participants, along with the 12 target words, thus making a total of 66 words. The participants’ task was to discriminate the words they had read during the training texts. To create the multiple-choice test, we employed a subset of the Collective Test of Reading Efficiency (Test *Colectivo de Eficacia Lectora*, *TECLE*) [24]. The number of items was reduced to 12, one for each of the target words. As in the Marín and Carrillo test, each item was made up of an incomplete sentence that lacks the last word, and 4 possible response options—only one of them was appropriate to finish the sentence. Also as in the Marín and Carrillo test, the foils were constructed so that two of them were pseudo words that differed only in one letter from the word target, and the third one was a phonologically similar word, but orthographically different from the word target. To build the picture-word matching test, we conducted a web search of pictograms, drawings and free distribution images that represented each of the target words. The test was made up of the 12 selected pictures, which were displayed along with the 12 target words in a random order. Participants were required to select the image corresponding to each word.

### Procedure

The training phase and the evaluation phase were carried out in groups with all students in the classroom. Before starting the training phase, participants were told that they would have to carefully read simple texts, trying to understand them. They were also told that the text could contain words that they would not know, and that they should guess the meaning from the context, while trying to understand the general meaning of the text. Then, they were randomly assigned to one of the two experimental sets and were asked to read their corresponding 9 texts during the training phase.

To avoid boredom or tiredness, the training phase comprised three sessions during three consecutive days, which were applied at the beginning of their regular classes (9:00 am) and in their everyday classroom. In each of the three days, the students read a fable, an expository text with science contents, and a text with math problems. As it was stated above, each text contained 4 of the 12 experimental words, so students read 3 times each of the 12 target words in their corresponding experimental condition (high vs. low contextual diversity). At the end of each text, students had to answer two questions of reading comprehension, with three possible answers of which only one was correct. They had unlimited time to read the texts and answer the comprehension questions. The presentation of the texts was randomized for each student, to minimize any potential primacy/recency effects in the evaluation phase.

On the fourth day, after completing the training phase, learning of target words was assessed through the different evaluation instruments previously introduced. To minimize carryover effects from one test to the other, the assessment was performed at two different times during the same day—the order of assessment was the same for high-contextual and low-contextual conditions. First, the students completed the free recall task, and this was followed by the recognition task—there was a five-minute break between them. Two hours later, they completed the multiple-choice test, followed by the picture-word matching test—there was a five-minute break between them.

## Results

The average of correct answers per session to the comprehension questions was 5.58 out of a total of 6 (range 5–6) which indicates that the students effectively read for comprehension. The mean percent correct and standard deviation of the four dependent variables for the high- and low-contextual diversity words, together with the 95% confidence intervals of the contextual diversity effect based on the by-subjects analyses (Table 3).

**Table 3:** Mean percent correct and standard deviations (in parentheses) of the four evaluation instruments for high contextual and low contextual diversity, together with the 95% confidence intervals for the contextual diversity effect.

	Free recall	Recognition	Multiple choice	Picture-word matching
High contextual diversity	6.06 (11.65)	48.48 (25.47)	74.75 (21.3)	50.51 (17.91)
Low contextual diversity	2.06 (5.52)	30.30 (21.43)	44.44 (21.11)	28.79 (17.32)
Contextual diversity effect	0.73–7.35	10.45–25.91	26.27–34.34	14.41–29.03

The participants’ and items’ average data were analyzed in separate Analyses of Variance (ANOVAs) with Contextual diversity (high diversity vs. low diversity) as a within-subject factor for each dependent variable. The dummy factor List (Set 1 vs. Set 2) was also included in the ANOVAs for the sole purpose of partialing out the error variance due to the two counterbalanced sets of items [25]. Results were clear: the mean scores for high contextual diversity words were greater than those for low contextual diversity words in all four dependent variables: free recall (6.1 vs. 2.1%, respectively), *F*1(1,31) = 5.95, *η*^*2*^ = .16, *p* = .02; recognition (48.5 vs. 30.3%, respectively), *F*1(1,31) = 23.14, *η*^*2*^ = .43, *p* < 0.001; *F*2(1,11) = 17.08, *η*^*2*^ = .61, *p* < .001; multiple choice (74.8 vs. 44.4%, respectively), *F*1(1,31) = 233.99, *η*^*2*^ = .88, *p* < 0.001; *F*2(1,11) = 39.96, *η*^*2*^ = .78, *p* < .001; and picture-word matching (50.5 vs. 28.8%, respectively), *F*1(1,31) = 36.01, *η*^*2*^ = .54, *p* < 0.001; *F*2(1,11) = 19.47, *η*^*2*^ = .64, *p* < .001. That is, the words presented in different contexts were learned and remembered more effectively than those that were presented in redundant contexts.

## Discussion

The current experiment examined whether contextual diversity—defined as the number of distinctive (non-redundant) contexts in which a new word appears—has a facilitative effect when learning new words in a classroom environment with developing readers (Grade 3 children). Results showed a facilitative effect of contextual diversity not only in a free recall task and a recognition task, but also in two tasks that tested the extent to which the children had acquired the meaning of the new words: a multiple-choice task which required the completion of sentences with targets out of three lexical distractors (orthographically or phonologically similar) and a task that required matching the nearly learned words with pictograms.

Taken together, the current findings are in line with the previous research that showed a facilitative role of contextual diversity across various tasks [8, 15–17, 21, 22]. While most of the previous studies focused on corpus-based measures that used a non-experimental manipulation (i.e., words of high and low contextual diversity were extracted from a word-corpus), here we directly manipulated contextual diversity. Furthermore, the mere count of the number of documents in a word-corpus (e.g., proportion of different contexts [e.g., films, documents] in which a given word appears) overlooks the potential semantic similarity between these documents (see [22] for discussion). For example, one could argue that the word *financial* typically occurs in movies/documents related to economy, so the semantic context of this word would be redundant. Recently, a number of laboratory experiments have directly manipulated contextual diversity in terms of the number of highly distinctive (non-redundant) contexts in which a new word appears during learning [17, 21, 22]. The present results, obtained in a highly ecological scenario (i.e., at the classroom during regular hour classes of Grade 3 children), paralleled previous experiments using a laboratory environment with adults. An important feature of the current study is that the facilitative effects of contextual diversity when learning new words occurred not only in two memory tasks, but also in two tasks that measured the acquisition of orthography/meaning of the new words (i.e., a multiple-choice test and a pictogram matching task) (see [17] for a similar effect with adult participants). Importantly, we should stress that the meaning of the new words was constant in all the contexts (i.e., the word *batracios* [the Spanish for batrachians] had exactly the same referential meaning across fables, natural science texts, and math exercises). Despite the importance of testing contextual diversity effects in developing readers, previous research has been very scarce and restricted to corpus-based stimuli (e.g., [13, 19]) rather than a direct manipulation of contextual diversity. As contextual diversity has proved to be a facilitative factor for adult readers during word learning, its effect should presumably be even greater for developing readers—note that their word representations are less consolidated [13]. In the present experiment, Grade 3 children did not have previous encounters with the target words, so the newly created word representations depended totally on the learning process in the classroom. This represents an ideal situation to evaluate how new lexical entries are naturally acquired and how the learning contexts modulate the strength and the structure of their lexical/semantic representations.

The Semantic Distinctiveness (SD) model [21] can readily accommodate the present findings because it assumes that the repetition of a word only produces a detectable modification in its memory strength when there is also a change in context. Each time a word is experienced in a new context, the model assesses how similar the current contextual information is to the memory representation of the word. If the contextual information is redundant with information already stored in the word’s memory representation, it is encoded at a lower weight and vice versa. When applied to the acquisition of new lexicon, this means that when a word is first experienced its memory representation is empty, hence it is encoded at maximal strength. Successive experiences with that word in other contexts will be coded with a strength that will depend on the similarity between this context and the word’s current lexical representation. Therefore, repeating a newly acquired word in highly distinctive contexts would produce much larger changes in its memory representation than repeating a newly acquired word in similar contexts.

To summarize, at a theoretical level, the present findings offer empirical support to those developmental models that implement a learning rule based on the different contexts in which a word has been experienced. This also leads to new questions about the concept of contextual diversity and its nature. Our results show that semantic diversity (i.e., the variety of the different contexts in which a word is experienced) facilitates word learning. A question for further research is whether the effects of contextual diversity have necessarily a semantic origin or whether may be partially due to more perceptual elements (e.g., visual background, speaker variability, among others). At an applied level, the presence of a facilitative role of contextual diversity in the classroom settings has important practical implications in education. To optimize the process of acquiring new words and concepts in the classroom, it is important to conduct a cross learning through different topics. A direct implication of the current findings is that educators should take contextual diversity into account in the curriculum design and in its implementation in the classroom. For example, it would be convenient to plan the learning of the essential lexicon in each educational stage by including it in activities from different subjects. More research is needed to test the effects of contextual diversity when learning abstract or polysemic words, new words in a second language—furthermore, the effects of contextual diversity could potentially be applied to other cognitive skills beyond word acquisition (e.g., math skills).

## Appendix

Example in Spanish and English of the three types of text in which the target word forraje (the Spanisg for *forage*) was inserted.

### Fábula: La liebre y la tortuga

En un bosque, había una liebre vanidosa que presumía de ser la más veloz y se burlaba de la tortuga por ser lenta. Un día, la tortuga, cansada de tantas burlas, retó a la liebre a una carrera.

Al poco de comenzar la carrera la liebre llevaba tanta ventaja que decidió parar a comer del forraje de un prado cercano, y a dar una cabezadita sobre el raigón de un gran árbol. Cuando despertó, varias horas después, miró hacia atrás y no vio a la tortuga. Echó de nuevo a correr, y a lo lejos vio la cinta de color bermejo que señalaba la meta, y a la tortuga que estaba a punto de cruzarla. La liebre corrió tanto como pudo, pero con los nervios tropezó con los guijarros del camino y perdió la carrera.

Ir poco a poco sin desviarse del camino puede llevarte más lejos que confiarse demasiado.

### Fable: The hare and the turtle

In a forest, a vain hare boasted of being the fastest and mocked the tortoise for being slow. One day, the turtle, tired of so many taunts, challenged the hare to a race.

Shortly after starting the race the hare was so advantageous that he decided to stop to eat from the forage of a nearby meadow, and to give a little head on the stump of a large tree. When he woke up, several hours later, he looked back and did not see the turtle. He ran again, and in the distance he saw the russet ribbon that marked the goal, and the turtle that was about to cross. The hare ran as far as he could, but with his nerves, he tripped on the cobblestones of the road and lost the race.

Going slowly without getting out of the way can take you further than trusting yourself too much.

### Texto de ciencias: Dentaduras de las distintas especies animales

La dentadura de las distintas especies animales está adaptada a su dieta, para poder consumir los alimentos existentes en su entorno.

Los mamíferos son los únicos vertebrados que mastican la comida para facilitar su digestión. El número, tamaño y posición de los dientes varía en función de su alimentación.

Los rumiantes, como el venado, tienen los molares grandes para poder masticar mejor el forraje del que se alimentan.

Los roedores comen, entre otras cosas, muchas simientes, por lo que tienen incisivos muy desarrollados, que no dejan de crecer porque se desgastan al roer cosas duras.

Por otro lado, los carnívoros tienen unos caninos grandes y afilados para poder desgarrar la carne de sus presas.

Muchos peces, como el tiburón, y algunos reptiles y anfibios también tienen dientes.

Sin embargo, las aves no tienen dientes, y en su lugar poseen un pico duro que usan para romper semillas y frutos.

### Science text: Dentures of different animal species

The dentition of the different animal species is adapted to their diet, in order to consume the existing foods in their environment.

Mammals are the only vertebrates that chew the food to facilitate its digestion. The number, size and position of the teeth varies depending on their feeding.

Ruminants, like the venison, have large molars to be able to chew better the forage from which they feed.

Rodents eat, among other things, many seeds, so they have very developed incisors, which do not stop growing because they wear out when biting hard things.

On the other hand, carnivores have large and sharp canines to tear the flesh of their prey.

Many fish, like the shark, and some reptiles and amphibians also have teeth.

However, birds do not have teeth, and instead have a hard beak they use to break down seeds and fruits.

### Problemas de matemáticas: Sumas, restas y multiplicaciones

Después de recoger la cosecha del grano en un campo de trigo, han segado el resto de la hierba y han hecho 23 fardos de forraje. Un granjero con su camioneta se lleva 14 fardos, ¿Cuántos fardos quedaran en el campo?

Cuando llega el verano y deja de llover, el río de mi pueblo se seca, y solo quedan 3 charcas donde nos bañamos. En cada una de esas charcas se encuentran nadando 6 batracios. ¿Cuántos hay en total?

En un pequeño prado había un rebaño de 35 ovejas comiendo hierba. De pronto, llegó una vulpeja y mató a 5 ovejas. ¿Cuántas quedaron al final?

En el pueblo de mi abuelo, mi tía Rosa tiene 4 vacas lecheras pastando en la dehesa. Su amiga Clara tiene 3 vacas más que ella. ¿Cuántas vacas tienen entre las dos?

### Math problems: Addition, subtraction and multiplication

After harvesting the grain in a field of wheat, they have mown the rest of the grass and made 23 bales of forage. A farmer with his truck carries 14 bales, how many bales will be left in the field?

When summer arrives and stops raining, the river of my town dries up, and there are only 3 ponds where we bathe. In each of these ponds are swimming 6 batrachians. How many are there in total?

In a small meadow was a flock of 35 sheep eating grass. Suddenly, a vulpine arrived and killed 5 sheep. How many were left?

In my grandfather's village, my Aunt Rosa has 4 dairy cows grazing in the meadow. Her friend Clara has 3 more cows than her. How many cows do they have between the two?

## Supporting information

S1 Dataset Data underlying the findings described in their manuscript. (XLSX)

S1 File Appendix A. Number of letters and Frequency of use per million words (from the EsPal database) of target words.
(DOCX)
