# Supplementary material for: Contextual diversity facilitates learning new words in the classroom
Source: PLoS One. 2017 Jun 6;12(6):e0179004. doi: 10.1371/journal.pone.0179004 (PMC5460874; doi:10.1371/journal.pone.0179004)
Supplement: S1 File — Number of letters and Frequency of use per million words (from the EsPal database) of target words. (DOCX) [file pone.0179004.s002.docx]

**Appendix A**

Number of letters and Frequency of use per million words (from the EsPal database) of target words.

| Target words | Length | Frequency per million |
| --- | --- | --- |
| Bermejo (russet) | 7 | 0.01 |
| Caninos (canines) | 7 | 0.02 |
| Dehesa (meadow) | 6 | 0.25 |
| Raigón (stump) | 6 | 0.02 |
| Venado (venison) | 6 | 0.23 |
| Batracios (batrachians) | 9 | 0.20 |
| Promontorio (promontory) | 11 | 0.09 |
| Forraje (forage) | 7 | 0.02 |
| Valvas (valves) | 6 | 0.00 |
| Guijarros (cobblestone) | 9 | 0.97 |
| Simientes (seeds) | 9 | 0.00 |
| Vulpeja (vulpine) | 7 | 0.00 |

Note: The approximate English translation is presented between brackets.
